# Effect of Low-Temperature Degradation, Ph-Cycling and Simulated Tooth Brushing on Surface Roughness, Topography, and Polish Retention of Yttrium-Stabilized Tetragonal Zirconia

**DOI:** 10.30476/dentjods.2022.93896.1744

**Published:** 2023-09

**Authors:** Foroogh Fadavi, Mahshid Mohammadi-Bassir, Nioosha Sarabi, Mohammad Bagher Rezvani, Siavash Jafari-Semnani, Maryam Rastegar Moghaddam, Hossein Labbaf

**Affiliations:** 1 Dept. of Operative Dentistry, School of Dentistry, Tehran University of Medical Sciences, Tehran, Iran; 2 Dept. of Operative Dentistry, School of Dentistry, Shahed University, Tehran, Iran; 3 Postgraduate, Dept. of Endodontics, School of Dentistry, Shahed University, Tehran, Iran; 4 Postgraduate, Dept. of Operative Dentistry, School of Dentistry, Shahed University, Tehran, Iran; 5 Dept. of Endodontics, School of Dentistry, Shahed University, Tehran, Iran

**Keywords:** Zirconia, Aging, Tooth brushing, Acidity, Dental polishing

## Abstract

**Statement of the Problem:**

Surface roughness of zirconia is an important parameter that determines the success of zirconia restorations. When zirconia surfaces are left rough, higher susceptibility to hydrothermal aging, plaque accumulation and color changes would occur. Therefore, polish retention of these restorations is considered as a challenge.

**Purpose:**

The purpose of this *in vitro* study was to determine the effect of hydrothermal degradation, pH- cycling, and simulated tooth brushing on surface roughness, topography, and polish retention of an yttrium-stabilized monolithic zirconia.

**Materials and Method:**

In this experimental study, 64 specimens of yttria-stabilized tetragonal zirconium oxide (20×4×2mm) were prepared (ZirKonzahn, Steger, Ahrntal). The specimens were wet- polished (standard polishing), and divided into 8 groups (n=8). Four control groups were assessed in non-aged condition while in 4 experimental groups the artificially ageing was done. Different finishing and polishing procedures were performed in 8 groups. The surface roughness values including mean surface roughness (Ra) and mean height of surface roughness (Rz) was measured by a profilometer. The results were analyzed using two-way ANOVA and Tukey’s HSD test (α=0.05). One representative specimen of each group was inspected under a scanning electron microscope (SEM) for assessment of surface topography.

**Results:**

The effects of surface treatments on Ra (*p*<.001) and Rz (*p*<.001) parameters were significant. Ageing had no significant effect on Ra (*p*=.086) and Rz (*p*=.067) values. Maximum Ra and Rz parameters were recorded following grinding (*p*<.001) and minimum values were recorded after glazing, which were significantly lower than the values in grinding group (*p*<.001). Polishing and glazing diminished the surface roughness (Ra) of ground zirconia similarly (*p*=.995).

**Conclusion:**

Aging had no significant effect on surface roughness of zirconia, irrespective of surface treatment type. Grinding yielded maximum surface roughness. Intra oral polishing yielded a surface roughness comparable to standard polishing and glazing.

## Introduction

Clinical applications of zirconia ceramics are increasing worldwide due to their favorable esthetics, optimal biocompatibility, dimensional and chemical stability, wear resistance, and high fracture toughness [ 1 - 2 ].

Zirconia is a polymorphic material with three different phases. Its monoclinic phase is stable by up to 1170°C temperature. It can transform to tetragonal phase, which is stable up to 2370°C. The cubic phase is stable up to 2680°C, which is the melting point of zirconia. Tetragonal-to-monoclinic phase transformation can also occur spontaneously over time due to body temperature and humidity, which is known as low temperature degradation. [ 1 - 3 ]. The mechanical properties of zirconia are related to its tetragonal to monoclinic phase transformation, which is induced by external compressive stresses and is associated with 3-5% volumetric expansion [ 3 ]. This expansion results in generation of internal stresses against cracking and serves as a factor that increases the resistance of zirconia against crack propagation [ 3 ]. 

The surface of zirconia crowns in the oral environment may undergo some changes due to the effect of saliva, pH alterations, mastication, and tooth brushing [ 3 ]. The wear of natural teeth opposing ceramic restorations is another matter of concern for dental clinicians, and evidence shows that the monolithic zirconia crowns cause less enamel wear of the opposing teeth compared with porcelain [ 3 ]. In the clinical setting, some intraoral adjustments are often required; these adjustments remove the glaze layer or polished surface and leaves a rough surface. This roughness can create an unaesthetic appearance, secondary caries, periodontal disease, excessive wear, and susceptibility to hydrothermal ageing [ 3 - 5 ]. 

In cases of occlusal adjustment especially after cementation, chairside intraoral polishing of restorations is effective and easy eliminating the need for additional laboratory steps [ 4 ]. For this purpose, dental clinicians should use a chairside ceramic polishing system, which is cost-effective and ensures long-term durability and success of restorations [ 5 ].

Surface roughness in zirconia restorations is an important parameter for determining the clinical success. Rougher surfaces accumulate more bacteria [ 6 - 8 ]. The mean surface roughness (Ra) and the mean height of the surface roughness profile (Rz) can be measured by profilometer [ 33 - 35 ]. The surface roughness threshold below which no bacterial accumulation occurs is reportedly 0.2 µm [ 9 - 10 ]. 

The pH alterations following the consumption of acidic foods and drinks or as the result of acid production by the oral microorganisms also affect the mechanical properties of zirconia. Ionic composition and buffering capacity of the saliva affect the properties of restorations in the oral cavity as well [ 9 , 11 ]. 

Tooth brushing is another important factor causing abrasion and surface roughness of restorations [ 21 ]. Although tooth brushing is imperative for proper oral hygiene, it can create mechanical and chemical stresses in restorative materials [ 22 ]. Tooth brushing can increase the surface roughness and cause discoloration in metal and ceramic crowns [ 23 ]. Duration of tooth brushing, the applied force, and the abrasiveness of toothpaste can all affect the surface roughness and gloss of restorations [ 24 - 25 ]. The effects of abrasion by tooth brushing on the surface characteristics of acryls, composite resins, and feldspathic ceramics have been previously studied. However, the cumulative effects of these factors on zirconia roughness and topography have not been well investigated [ 21 , 26 ].

Thus, this study aimed to assess the effects of low temperature degradation, pH-cycling, and tooth brushing on surface roughness, topography, and polish retention of monolithic zirconia. The first null hypothesis was that different surface treatments would have no significant effect on surface roughness (Ra and Rz values). The second null hypothesis was that aging by low-temperature degradation, pH-cycling, and simulated tooth brushing would have no significant effect on Ra and Rz surface roughness values of zirconia restoration.

## Materials and Method

Sixty-four bar-shaped specimens (20×4×2mm) were fabricated from presintered monolithic zirconia blanks (ZirKonzahn Steger, Ahrntal, Italy) using a copy-milling machine (Amanngirrbach, Austria). For the fabrication of bar-shaped zirconia specimens, first, a composite model measuring 2×4×20mm was fabricated and its dimensions were measured by a digital caliper (Mitutoyo, Japan) with 0.1mm accuracy. Then, the composite model was placed in the holder of scanner and milled by taking into account the shrinkage rate reported by the manufacturer [ 26 - 28 ]. The zirconia specimens were then sintered at 1450°C according to the manufacturer’s instruction (Amanngirrbach, Austria) (Table 1). Afterwards, all the sintered specimens were wet-polished for 15 seconds with 600-,800-, and 1200 –grit silicon carbide papers (Struers A/S) under 10-N load using a grinding/ polishing machine (Phoenix Beta grinder/ polisher; Buehler) at a speed of 300 rpm as standard polish [ 28 ] (Table 2). Then the specimens randomly assigned to 8 experimental groups (n=8).

**Table 1:** Firing chart for zirkonzahn

Start temperature	Drying time	Preheating time	Heating rate	Hold time	Vacuum on	Vacuum off	Vacuum level	Cooling
300°C	2 min.	6 min.	25-55°C	2-3 min.	400°C	820°C	Max.	3-5 min.

**Table 2:** Materials used in this study

Material	Definition	Main Composition	Manufacturer
Zirkonzahn	Yttrium partially stabilized zirconia (Y-TZP)	ZrO_2_ w%: Main component, Y_2_O_3_ w%:4.95~5.26, Al_2_O_3_ w%:0.15~0.35, SiO_2_ w%:Max. 0.02, Fe_2_O_3_ w%:Max. 0.01, Na_2_O w%:Max.0.04	Steger, Ahrntal, Italy
Grinding paper	Polishing discs	Silicon carbide Grit size: 600, 800, 1200	Germany
Diamond Bur	Cylindrical blue-yellow band diamond bur	Diamond Grit size:108-120µ Code: ZD881 (ISO, 806.314.141…016)	D+Z, Germany
Panasil	Heavy body	Addition silicon	Kettenbach, Germany
EVE polishing kit	Medium rubber (green yellow)	Silicon dioxide matrix medium and fine grit diamond abrasive	Ernst Vetter GmbH, Germany
	Fine rubber (pink yellow)		

**Figure 1:**
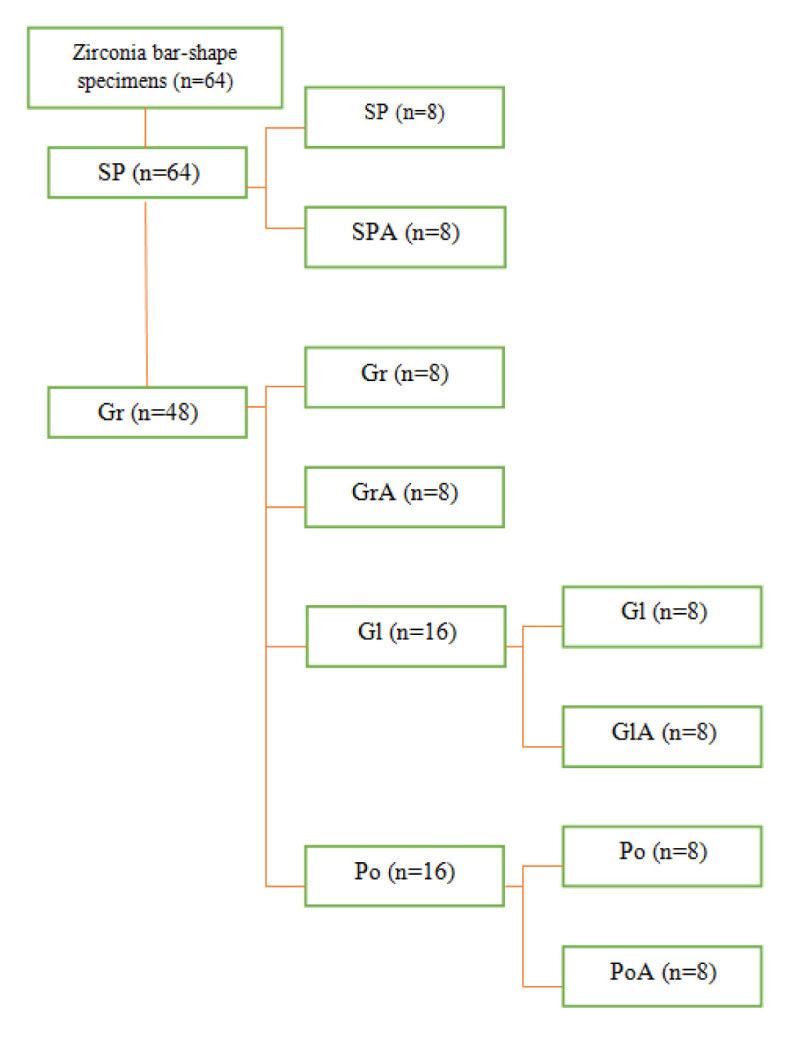
Flowchart of groups of specimens (SP: Standard Polish, Gr: Grinding, Gl: glazing, Po: Polishing with an intraoral zirconia polishing system, A: Ageing)

In Group SP, the specimens were standard polished without any surface treatments. In Group SP-A, the specimens underwent standard polishing and artificial ageing. In Group Gr, they were wet-ground with blue–yellow band diamond (108 to 120 µm) rotary instrument (D+Z, Germany) in a forward-backward sweeping motion under water coolant. A new diamond rotary instrument (DRI) was used for every 5 specimens. Grinding and polishing were performed by the same operator (F.F.). In Group Gr-A, the grinding of the specimens was performed as group Gr, and then they underwent ageing.IN, Group Gl, the grindings of the specimens were performed as group Gr and then they were overglazed according to the manufacturer instructions. The overglazing powder was mixed with the glaze liquid and applied in a very thin layer and then fired at 820ºC for two minutes (Zirkon glaze and Zirkon ICE Stain Liquid; ZirKonzahn, Germany). In Group Gl-A, the surface grinding and glazing was performed as group Gl, and then they underwent ageing. In Group Po, the grindings of the specimens were performed as group Gr and then they underwent wet polishing with an intraoral zirconia polishing system (Diacera; EVE, Germany) in two- steps with green (medium) and pink (fine) polishing rubbers for 60 seconds. Polishing was performed with low-speed handpiece (NSK, Japan) in a forward-backward sweeping motion under water coolant. In the last group (Group Po-A), the surface grinding and polishing was performed as Group Po and then they underwent ageing (Figure 1).

The specimens were then debrided in ultrasonic bath (Dentine XL, Iran) containing distilled water and were immersed in 100% ethanol for 1 minute, followed by rinsing and air-drying. 

Hydrothermal ageing was performed by low temperature degradation according to ISO13356 at 134°C at 2 bar vapor pressure for 40 hours. [ 29 ] For pH- cycling, the specimens were immersed in 5 mL of demineralizing solution and incubated at 37°C for 18 hours. The demineralizing solution was an acidic solution containing 2mM calcium and 2mM phosphate in a buffering solution of 0.74 mM acetate with a pH of 3.4. The mineralizing solution (artificial saliva) contained 1.5 mM calcium, 0.9 mM phosphate (in 20 mM Tris buffer), and 150 mM potassium chloride (hydroxyl methyl aminomethane) at a pH of 7. These cycles were repeated for 15 times [ 11 , 18 - 20 , 30 ]. Finally, the specimens were rinsed in deionized distilled water and incubated at 37°C in 100% humidity.

For simulated tooth brushing, Crest V Complete toothpaste (Gross Gerau, Germany) and water were weighed by a digital scale and mixed in 1:3 ratios in a graded beaker to obtain a homogenous suspension [ 8 , 31 - 32 ]. This suspension was poured into the cylinders in which, the specimens and toothbrush were located such that the surface of specimens were completely covered with the toothpaste suspension. The specimens were brushed by the automatic brushing machine (V8 Brushing Machine; Dorsa, Iran) with back-and-forth motion of soft bristles (Classic 411VSA; GUM). The head of toothbrush was perpendicular to the specimen surface and moved at a speed of two strokes per second for 10,000 back and forth cycles.

The surface of specimens was scanned by a profilometer (Hommel Werke T8000, Germany). For this purpose, the profilometer was first calibrated, and the assessments were performed in all eight groups. Ra and Rz were measured and recorded in micrometers (µm) [ 33 - 35 ]. Three different measurements were made at three points on each specimen surface with 4 mm distance from each other with the crosshead speed of 0.5 mm/second. The mean Ra and Rz of the three measurements were calculated and reported in micrometers (µm). After profilometry, one specimen of each experimental group with the closest surface roughness value (Ra) to the mean value of the respective group was selected for scanning electron microscopy (SEM; Cam scan MV 2300). The selected specimens were gold-coated and inspected under an electron microscope at 700× and 3000× magnifications, and topographical changes were evaluated.

Data were analyzed using SPSS version 25 (SPSS Inc.). The mean, standard deviation, minimum and maximum values of Ra and Rz were calculated and reported for the eight groups. The Kolmogorov-Smirnov test confirmed normal distribution of data. Two-way ANOVA test was applied to assess the effect of surface treatment and aging on surface roughness parameters. Pairwise comparisons were carried out by Tukey’s test. The level of significance was set at 0.05.

## Results

### Surface roughness

Table 3 shows the mean, standard deviation, minimum, and maximum values of surface roughness in eight experimental groups. Two-way ANOVA revealed significant differences in the mean Ra and Rz values between the eight groups (*p*<0.001 for Ra and *p*<0.001 for Rz). However, ageing had no significant effect on Ra (*p*=.086) and Rz (*p*=0.067) values. Considering the presence of significant differences in the mean Ra and Rz values in different groups, the Tukey’s post-hoc HSD test was applied for pairwise comparisons.

**Table 3:** Mean, standard deviation, minimum and maximum values of surface roughness

Group	Surface roughness variable	Minimum	Maximum	Mean	Std. deviation
SP	Ra1	0.09	0.14	0.111a	0.018
	Rz1	0.88	1.19	1.07A	0.101
SP-A	Ra2	0.1	0.15	0.126a	0.018
	Rz2	0.9	1.26	1.14A	0.119
Gr	Ra1	1.36	2.86	2.13b	0.612
	Rz1	3.27	4.42	3.82B	0.364
Gr-A	Ra2	1.75	3.43	2.62b	0.651
	Rz2	3.69	4.93	4.19B	0.379
Gl	Ra1	0.08	0.1	0.089a	0.008
	Rz1	0.64	0.71	0.676C	0.026
Gl-A	Ra2	0.09	0.11	0.095a	0.008
	Rz2	0.65	0.72	0.683C	0.026
Po	Ra1	0.09	0.14	0.104a	0.019
	Rz1	0.79	1.15	0.965A	0.123
Po-A	Ra2	0.09	0.13	0.111a	0.015
	Rz2	0.81	1.27	1.033A	0.165

SP: Standard polishing; A: Aging; Gr: Grinding; Gl: Glazing; Po: Polishing; Different letters indicate significant differences (lowercase letters for Ra and uppercase letters for Rz)

Table 4 presents pairwise comparisons of the groups regarding Ra. The results showed a significant difference in mean Ra values between Gr (2.86µm), SP (0.111µm), Gl (0.089µm), and Po (0.104µm) groups (*p*< 0.001) such that grinding significantly increased the surface roughness compared with standard polishing, glazing, and polishing with Diacera system. After polishing with Diacera system, the Ra value (0.104µm) was not significantly different from the corresponding value in Group Gl (Ra=0.089µm) and Group SP (*p*= 1.0) therefore, glazing and polishing with Diacera system similarly diminished the mean surface roughness (*p*= 0.995). 

**Table 4:** Pairwise comparisons of the groups regarding Ra

Group (I)	Group (J)	Mean difference	Std. error	*P* Value
SP	Gr:	2.8963	0.07343	0.001
	Gl:	0.4281	0.07343	0.001
	Po:	0.1081	0.07343	0.46
Gr:	Gl:	3.3244	0.07343	0.001
	Po:	3.0044	0.07343	0.001
Gl:	Po:	0.32	0.07343	0.001

SP: Standard polishing, Gr: Grinding, Gl: Glazing, Po: Polishing

Table 5 presents pairwise comparisons of the groups regarding Rz. A significant difference was noted in mean Rz values between SP (1.07 µm) compared with Gr (3.82µm), Gl (0.676µm), and Po (0.965µm) groups (*p*< 0.001) such that grinding created a rougher surface with more irregularities compared with standard polishing, glazing, and polishing with Diacera system. However, Glazing created more homogenous surface than polishing with Diacera system (*p*<0.0010).

**Table 5:** Pairwise comparisons of the groups regarding Rz

Group (I)	Group (J)	Mean difference	Std. error	*P* Value
SP	Gr:	2.8963	0.07343	0.001
	Gl:	0.4281	0.07343	0.001
	Po:	0.1081	0.07343	0.46
Gr:	Gl:	3.3244	0.07343	0.001
	Po:	3.0044	0.07343	0.001
Gl:	Po:	0.32	0.07343	0.001

SP: Standard polishing, Gr: Grinding, Gl: Glazing, Po: Polishing

Two-way ANOVA revealed that ageing had no significant effect on Ra (*p*= 0.086) and Rz (*p*= 0.067) values. In total, maximum Ra and Rz values before and after ageing were noted in grinding group whereas glazing group showed minimum surface roughness values (Table 3).

### Surface topography

**Figure 2:**
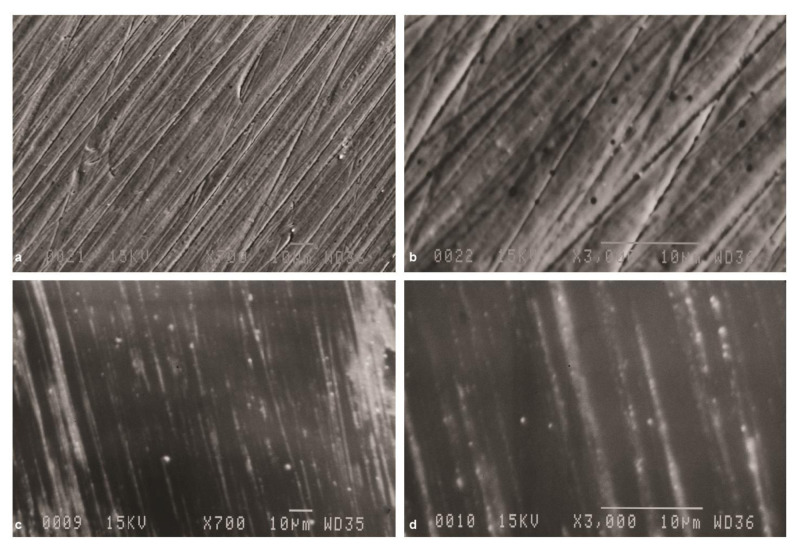
Scanning electron microscope (SEM) micrographs of zirconia specimen after standard polishing (group SP), **a:** Original magnification 700×, multidirectional shallow grooves are seen after polishing with silicon carbide papers, **b:** Original magnification 3000×, parallel unidirectional lines are seen. Original magnification 700×, **c:** SEM micrograph of zirconia specimen after standard polishing and aging (group SP-A), **d:** Original magnification 3000×, surface smoothness is increased but aging could not eliminate shallow grooves created by standard polishing

In Group SP, inspection at 700× magnification (Figure 2a) revealed shallow multidirectional lines due to the use of silicon carbide discs for standard polishing. Apparently, standard polishing could not completely eliminate the surface irregularities. At 3000× magnification (Figure 2b), parallel unidirectional lines were observed and some residual abraded particles were noted on the surface. SEM micrographs in Group SP-A revealed that aging process could not significantly affect the surface roughness (Figures 2c-d).

**Figure 3:**
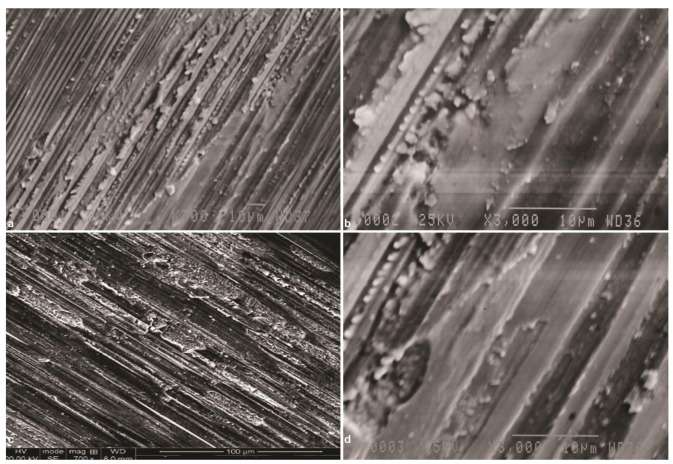
Scanning electron microscope (SEM) micrographs of zirconia specimen after grinding (group Gr), **a:** Original magnification 700×,multiple deep unidirectional scratches and some debris created by surface preparation are seen, **b:** Original magnification 3000×, deep unidirectional scratches formed by random arrangement of abrasive particles on DRI can be seen, original magnification700×, **c:** SEM micrograph of zirconia specimen after grinding and aging (group Gr-A), **d:** Original magnification 3000×,aging could not decrease surface roughness

In Group Gr, inspection at 700× magnification (Figure 3a) revealed deep unidirectional scratches and residues of debris adhered to the surface in the form of a smear layer. Plastic deformation of material was clearly visible. At 3000× magnification (Figure 3b), the same pattern was observed more clearly. SEM micrographs in Group Gr-A, revealed that aging process could not significantly decrease the surface roughness (Figures 3c-d). 

**Figure 4:**
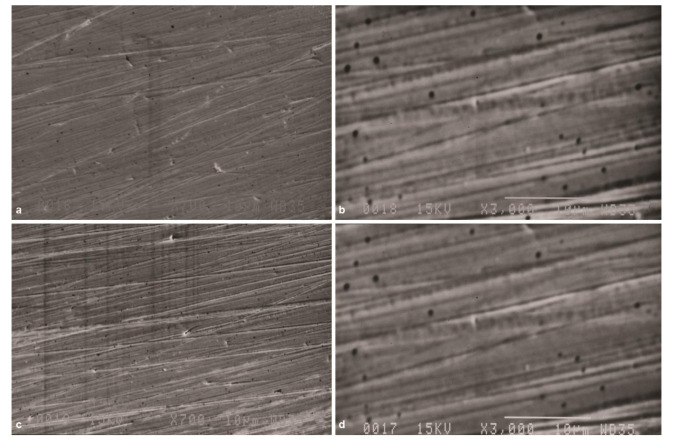
Scanning electron microscope (SEM) micrographs of ground zirconia specimen after glazing (group Gl), **a:** Original magnification 700×,the specimen surface is relatively smooth and some unidirectional lines are seen, **b:** Original magnification 3000×,small voids indicate incomplete coverage of defects by glaze layer, **c:** Original magnification 700×, SEM micrograph of ground zirconia specimen after glazing and aging (group Gl-A) aging could not adversely affect the surface roughness, **d:** Original magnification 3000×, glazing could not eliminate shallow grooves created by standard polishing

In Group Gl, SEM micrographs revealed relative smoothness of the surface (Figures 4a-b). However, the glaze layer could not completely fade the deep scratches created during grinding of specimens. Although the scratches were not completely filled, smoothness of the surface was due to the presence of a glaze layer on the surface. However, some small voids were noted on the surfaces, which indicated incomplete coverage by the glaze layer. SEM micrographs in Group Gl -A revealed that ageing process could not significantly affect the surface roughness (Figures 4c-d). 

**Figure 5:**
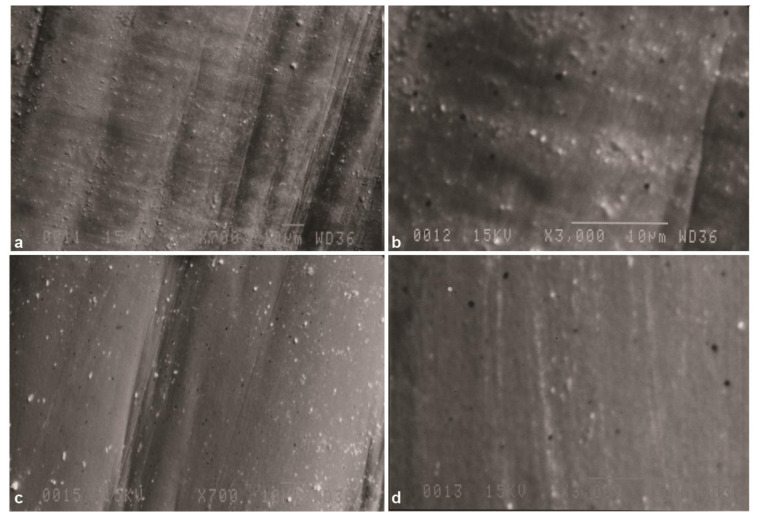
Scanning electron microscope (SEM) micrographs of ground zirconia specimen after polishing with Diacera system (group Po), **a:** Original magnification 700×. The surface topography is smoother than standard polishing, **b:** Original magnification 3000×. Deep grooves are not eliminated and some residues of rubber polisher is seen, **c:** Original magnification 700×. SEM micrograph of ground zirconia specimen after polishing with Diacera system and aging (group Po-A), **d:** Original magnification 3000×, aging could not significantly affect the surface roughness

In Group Po, SEM micrographs revealed significantly smoother surface than group Gr (Figures 5a-b). However, deep scratches created during grinding were not omitted completely. The surface topography of specimens in this group was slightly smoother than that in SP group. However, some residues of polishing rubber were seen on the surface. SEM micrographs in Group Po-A revealed that ageing process could not significantly affect the surface roughness (Figures 5c-d). 

## Discussion

The results of this study revealed that the effects of surface treatments on Ra (*p*< 0.001) and Rz (*p*< 0.001) parameters were significant. This result did not support the first null hypothesis that different surface treatments would have no significant effect on surface roughness (Ra and Rz values). Our findings showed that ageing had no significant effect on Ra (*p*=0.086) and Rz (*p*< 0.067) values. Thus, our results supported the second null hypothesis that ageing would have no significant effect on Ra and Rz. Maximum Ra and Rz parameters were recorded following grinding (*p*< 0.001) whereas minimum values were recorded after glazing (*p*< 0.001). 

In this study, the surface roughness of all specimens was first standardized by sequential use of course-to-fine grit silicon carbide papers in a standard polishing device [ 26 , 36 ]. Standard polishing was performed for all specimens to eliminate surface irregularities and bevel the chipped corners of specimens [ 2 ]. In addition, one calibrated operator performed grinding of all specimens for the purpose of standardization [ 4 ]. The pH-cycling and low temperature degradation were performed under standard controlled conditions, and simulated tooth brushing was performed according to a previously tested protocol [ 1 , 11 ]. A method for simulating the pH variations in oral environment is not well established. Some researcher used gastric acid juice and HCL with different pH values (1.2 to3.8) for different periods (6 to96 hours) [ 12 - 15 ].

Alnasser *et al*. [ 16 ] reconstructed an acidic condition such as bulimia nervosa and gastroesophageal reflux disease, whereas Turp *et al*. [ 17 ] stored the zirconia specimens in three solutions with pH values of 3.5, 7.0 and 10. In the present study for simulation of a high cariogenic challenge, the specimens were cycled through an alternating demineralizing and remineralizing regimen [ 18 - 20 ]. For assessment of surface roughness, the Ra and Rz parameters were measured according to ISO4287 and previous studies [ 5 , 31 , 37 - 38 ]. Ra is commonly measured for assessment of surface roughness of zirconia. However, Queiroz *et al*. [ 38 ] showed that Ra with 2D images of the surface provides limited information, which often leads to misinterpretation of surface roughness. Moreover, Ra alone cannot identify the differences between the superficial irregularities (peaks and valleys) [ 38 ]. Thus, the Rz parameter should also be necessarily measured for a more accurate assessment of surface roughness. In fact, Rz indicates the mean distance between 10 most prominent and deepest points on the surface and indicates whether the surface roughness is homogenous [ 39 ]. Homogeneity (evenness) of the restoration surface is important between two restorations with similar Ra values, the one with lower Rz value would have superior clinical efficacy and cause lower wear of the opposing teeth [ 39 ]. In this regard, glazing created more homogenous surface (0.676 µm) than polishing with Diacera (0.965µm) system (*p*< 0.0010). In addition, a DRI with blue-yellow band and 108 to 120 µm particles was used to roughen the surface of all specimens to simulate occlusal adjustment in the clinical setting. This DRI is commonly used for occlusal adjustment in the clinical setting and has been specifically designed by the manufacturer (D + Z) for this purpose [ 27 ]. Iseri *et al*. [ 36 ], used a DRI with 150 µm particles on a high-speed handpiece whereas Subasi *et al*. [ 40 ], used 110µm DRI in dry condition for this purpose. The current results revealed a significant difference in the mean Ra and Rz values between the Gr and SP groups such that grinding significantly increased both surface roughness parameters. This result was expected since diamond is a super abrasive material and has a higher abrasive property than silicon carbide discs used for standard polishing [ 41 ]. 

Glazing is a commonly used method to smoothen the rough surfaces after occlusal adjustment. Glazing minimizes plaque accumulation and yields a glossy surface resembling natural tooth. In this study, glazing significantly decreased the mean Rz value compared with polishing (*p*< 0.001). Considering the potential of the glaze layer in filling the surface crack, the lower Rz value was predictable. The use of Diacera polishing system after grinding yielded a mean surface roughness value comparable to the value in the SP group. The manufacturer does not disclose any information regard ing the size of diamond particles present in the rubber discs of the Diacera polishing system. However, according to textbook, diamond particles in polishing rubber discs are fine (<20µm) [ 41 ]. Thus, they seem to have the same effect on the zirconia surface as the fine silicon carbide paper discs. An interesting finding of this study was slight reduction of the mean surface roughness after glazing, which was not significant. SEM micrographs revealed smoother surface in both groups after glazing, although deep grooves were not omitted. The Diacerapolishing system resulted in acceptably smooth surface with a mean surface roughness (Ra=.104 µm) lower than the acceptable value by the patients (0.2 µm) [ 9 ]. In this study, the mean Ra and Rz parameters after grinding were significantly higher than the corresponding values in other groups and minimum values were noted after glazing. This result was expected considering the high hardness and abrasiveness of diamond [ 41 ]. Moreover, the size of diamond particles in this study was 108-120 µm, which was very larger than the size of particles in polishing discs [ 27 ]. Diamond particles remove zirconia by creating small fractures. Application of DRI causes superficial damage and creates debris [ 42 ]. The increase in surface roughness due to grinding has been previously evaluated. [ 43 - 44 ]. In the present study, Diacera polishing system yielded surface roughness values (Ra) comparable to the standard polishing and glazing (p>0.05). Thus, use of polishing system after occlusal adjustment in the office is logical and cost-effective. The efficacy of finishing and polishing systems depends on the structure and mechanical properties of the substrate, size of abrasive particles, and physical properties of the binder, which can be rigid or elastic [ 45 ].

Mohammadi-Bassir *et al*. [ 27 ] evaluated the effects of coarse grinding, over-glazing, and two polishing systems on surface roughness of zirconia. They showed that polishing with two intraoral polishing systems namely Busch and Meisinger (Busch and co, Germany) decreased the surface roughness to the level of over-glazing. Their observations were in line with the results of the current study, although different systems were used in the two studies. Hmaidouch *et al*. [ 4 ] measured the surface roughness of full-contour zirconia crowns following grinding and polishing with intraoral polishing kits and showed that fine polishing of zirconia yielded surface roughness values comparable to the glazing method. They explained that surface polishing of zirconia would eliminate defects caused by grinding and result in more favorable distribution of surface defects. On the other hand, presence of small voids in the glaze layer can explain the similarity of surface roughness after polishing and glazing. 

Preis *et al*. [ 37 ] assessed the surface topography of monolithic zirconia specimens after grinding and polishing, and found that grinding significantly increased the surface roughness of sintered zirconia whereas polishing caused a significant reduction in surface roughness, which was in agreement with the current findings. Glazing is reportedly the best surface treatment for ceramics, and the surfaces prepared by DRI should be glazed again before cementation to maximize restoration smoothness and durability. Intraoral polishing is an acceptable alternative to glazing after occlusal adjustment [ 4 , 27 , 37 , 46 ]. 

Our results revealed that aging had no significant effect on Ra whereas its effect on Rz was significant. Khayat *et al*. [ 47 ] assessed the zirconia surface roughness after grinding and polishing before and after aging. They showed that grinding increased the surface roughness but aging had no significant effect. Their results were in agreement with our findings. Candido *et al*. [ 31 ] assessed the effects of 10 years of tooth brushing on surface roughness of zirconia ceramics and showed that mechanical stress of tooth brushing with toothpaste did not cause a significant change in surface roughness; their results were in agreement with our findings. However, we assessed the effects of tooth brushing along with pH -cycling and low-temperature degradation. In general, tooth brushing with the conventional toothpastes can increase the surface roughness of zirconia in absence of glaze layer within 10 to 12 years [ 48 ]. 

In the current study, SEM examination in Group Gr revealed deep unidirectional scratches, which were in the same direction as the direction of DIR movement. These findings were supported by the results of profilometry that showed maximum roughness in this group. Similarly, Preis *et al*. [ 37 ] reported that grinding roughened the zirconia surface, and deep grooves were observed under SEM that had become smoother by polishing. Their results were in line with our findings. SEM revealed that polishing could not completely eliminate the grinding scratchers, which was in agreement with the results of previous studies [ 37 , 49 ]. 

Aging had no significant effect, and maximum roughness, irregularities, and cracks were noted in groups Gr and Gr-A. In Gl and Gl-A groups, the surfaces were smooth, due to the presence of glaze layer on the surface. Aging could not eliminate the glaze layer either. In Group SP, multidirectional lines were noted on the surface due to the use of polishing discs in two perpendicular directions. The Diacera polishing system yielded a smooth surface with some voids. 

This study had some limitations. It had an *in vitro* design and use of controlled parameters such as number of tooth brushing strokes and pH cycles could not well simulate the dynamic oral environment, pH alterations, variable masticatory forces, and presence of saliva and bacteria. In addition, in our study, flat surfaces of zirconia were investigated, which might have different results than non-flat and prominent surfaces of zirconia restorations in the oral environment. [ 50 ]. No standards have been set for perfect simulation of clinical setting [ 50 ]. Thus, future studies should assess the effect of different tooth brushing conditions and different pH - cycles on surface roughness of different ceramic specimens.

## Conclusion

Within the limitations of this study, the results showed that aging by low temperature degradation, pH- cycling, and tooth brushing had no significant effect on surface roughness of zirconia, irrespective of surface treatment. Maximum surface roughness of zirconia was noted following grinding. The Diacera polishing system yielded a surface roughness comparable to standard polishing and glazing.

## Conflict of Interest

The authors declare that they have no conflict of interest.
